# Identification of the homozygous truncating mutation in *CNTD1* as a novel genetic cause of diminished ovarian reserve

**DOI:** 10.1016/j.gendis.2025.101900

**Published:** 2025-10-24

**Authors:** Liwei Sun, Yi Chen, Keya Tong, Weiwei Liu, Bei Liu, Yifan Wang, Guoning Huang, Jingyu Li

**Affiliations:** Chongqing Key Laboratory of Human Embryo Engineering and Precision Medicine, Center for Reproductive Medicine, Chongqing Health Center for Women and Children, Women and Children's Hospital of Chongqing Medical University, Chongqing 400016, China

**Keywords:** *CNTD1*, Diminished ovarian reserve, Novel genetic cause, Splicing mutation, Whole-exome sequencing

## Abstract

Diminished ovarian reserve (DOR) is one of the leading causes of infertility, which accounts for approximately 10% of women seeking fertility treatment. However, their genetic etiology and pathogenesis are largely unknown. Recently, cyclin N-terminal domain containing 1 (*CNTD1*) was reported to be critical for meiosis in female mice. However, no *CNTD1* mutation has been reported to be associated with reproductive diseases in humans. Here, we firstly identified *CNTD1* mutation in a DOR patient. The homozygous *CNTD1* splicing mutation (NM_173478.3: c.823-2A > G) was identified in a DOR patient by whole-exome sequencing. The pathogenic effect of the identified *CNTD1* splicing mutation was investigated by sequencing the transcript from the patient's primary leukocytes and minigene assay. A CRISPR/Cas9-mediated *Cntd1* knockout mouse line was generated to investigate its role in ovarian function. The pathogenic mechanism of the identified *CNTD1* mutation was further verified by functional studies. As a result, minigene assay and direct transcript sequencing from the patient revealed that this splicing mutation induced aberrant exon skipping. The homozygous truncating mutation in *CNTD1* result in the production of a C-terminally truncated protein that cannot interact with its essential meiosis partner of proline-rich protein 19 (PRR19). *Cntd1* knockout mice were characterized by dramatically reduced size of ovaries and prematurely depleted follicular pools, which indicated its role in female fertility. In conclusion, this study is the first to identify *CNTD1* as a novel genetic cause for DOR patients and suggests the essential role of *CNTD1* in human reproduction.

## Introduction

Ovarian reserve is a primary determinant of the reproductive potential in the female lifespan.^1^ Diminished ovarian reserve (DOR) is one of the leading causes of infertility, which accounts for approximately 10% of women seeking fertility treatment.^2^ DOR is an ovarian disorder characterized by a reduced number or quality of oocytes in women of reproductive age, and it poses a conundrum for assisted reproductive technology due to poor ovarian response and high cancellation rates.^3^ Genetic factors associated with ovarian reserve have been revealed to be responsible for DOR or premature ovarian failure, such as growth differentiation factor 9 (*GDF9*).^4^^,^^5^ Nevertheless, their etiology and pathogenesis are still vague.^6^ Therefore, identifying novel causative genetic mutations for these disorders is essential to elucidate the etiology of ovarian dysfunction, and also benefits the diagnosis and genetic counselling of the DOR patients.

Cyclin N-terminal domain containing 1 (*CNTD1*), an ortholog of crossover site-associated-1 (COSA-1) in *Caenorhabditis elegans*, is indispensable for meiosis.^7^ Disruption of *Cntd1* in male mice is sterile, resulting in severe meiotic disruption in late prophase I spermatocytes, and significantly decreased testis size, and no epididymal spermatozoa were observed.^8^, ^9^, ^10^ In a recent study, *Cntd1* was reported to be crucial for female meiosis and the ovarian reserve establishment in mice. Lack of *Cntd1* in female mice exhibited severe sterility, and depleted follicle pool in ovaries.^11^ Despite demonstrating a conserved role of *Cntd1* during male and female fertility in mice, no *CNTD1* mutation has been reported to be associated with reproductive diseases in humans.

Here, we firstly reported *CNTD1* mutation that is responsible for human DOR, suggesting *CNTD1* mutation as a novel genetic cause of DOR in humans. We verified the pathogenic effects of the identified *CNTD1* mutation by functional studies in cultured cells and a knockout mouse model. Our findings lay the foundation for genetic diagnoses and counselling in DOR patients, and highlight the essential role of *CNTD1* in human reproductive diseases.

## Materials and methods

### Participants and whole-exome sequencing of genomic DNA

Patients presented with DOR, and the females with normal ovarian reserve were recruited. A total of 435 infertile patients were recruited in our study, and whole-exome sequencing was performed in all patients, including 43 patients with DOR and 392 patients with normal ovarian reserve. The diagnostic criteria for DOR were based on ovarian reserve testing, including measurement of antral follicle count (AFC) below 5 by ultrasound, decreased level of anti-müllerian hormone (AMH) under 1 ng/mL, or increased levels of basal follicle-stimulating hormone (FSH) above 10 IU/L. Genomic DNA was extracted and purified from the recruited patients based on the operation instructions (AmCare Genomics Lab, Guangzhou, China). Sequencing libraries were generated and sequenced with the read depths of 100 × (MGISEQ-2000, Shenzhen, China). Genetic testing was performed according to the Declaration of Helsinki. This study was approved by the ethics committee of the Women and Children's Hospital of Chongqing Medical University (2020-RGI-04, 22 June 2020). The written informed consents were obtained from all participants.

### Analysis of sequencing data

The sequencing reads were aligned to the human reference genome (hg19, February 2009, build 37.5). The annotation scope of variant types includes mutations in each exon, as well as mutations within 10 base pairs upstream and downstream of each exon, including missense, nonsense, synonymous, frameshift, in-frame, and splice site mutations. Pathogenicity of the identified variants was evaluated based on population databases, including the gnomAD (Genome Aggregation Database) and OMIM (Online Mendelian Inheritance in Man). The conservation and pathogenicity of the variants were predicted by SIFT,^12^ Polyphen2,^13^ MutationTaster,^14^ FATHMM,^15^ and other prediction software. The guideline from the American College of Medical Genetics and Genomics (ACMG) was followed during the evaluation of identified variants.^16^ The screened candidate variants were confirmed in the whole pedigree by Sanger sequencing.

### Cell culture

Dulbecco's modified Eagle's medium (DMEM) (6123130; Gibco, USA) supplemented with 10% fetal bovine serum (A5670701; Gibco, USA) and penicillin/streptomycin (100 U/ml) at 37 °C in 5% CO_2_ was used for the HEK-293T cell culture.

### Real-time quantitative PCR

The total RNA was extracted by Trizol (15596026CN; Thermo Fisher Scientific, USA) according to the standard instructions from the manufacturer. cDNA was generated by PrimeScript RT Master Mix (RR036; Takara, Kusatsu, Japan) during reverse transcription. In case there was DNA contamination, the RNase-Free DNase Set (79254; Qiagen, Hilden, Germany) was applied. For expression pattern analysis, the single-cell RNA-sequencing data of human oocytes and granulosa cells were obtained from a previously published database (GSE107746),^17^ and quantitative PCR was performed by the use of premix (RR820; Takara, Kusatsu, Japan). The CFX96 Real-Time System (Bio-Rad, Hercules, California, USA) was applied.

### Immunofluorescence analysis

For tissue sections, mice were anesthetized, and the ovarian tissue was fixed with 4% paraformaldehyde. Ovaries were embedded in Tissue-Tek O.C.T. compound (Leica, Germany) and frozen at 80 °C after fixation in 4% paraformaldehyde. Sagittal sections were cut using a Leica CM1950 microtome at a thickness of 8 μm. The primary antibody of anti-CNTD1 (Proteintech, 24854-1-AP) was used for overnight incubation at 4 °C.

### Generation and genotyping of *Cntd1* knockout mouse

*Cntd1* knockout mouse lines were generated using CRISPR/Cas9 genome editing.^18^ The guide RNAs (gRNAs) were designed using the online platform at http://crispr.mit.edu/. All animal experiments were performed following the protocols of the Institutional Animal Care and Use Committee at Women and Children's Hospital of Chongqing Medical University.

### Histological analysis

For histological analysis, mouse ovaries were fixed with 4% formaldehyde in phosphate-buffered saline at 4 °C overnight, dehydrated in an ethanol gradient, embedded in paraffin, and sectioned into 5 μm sections. The obtained sections were then deparaffinized, rehydrated, and stained with hematoxylin/eosin following standard procedures.

### Immunoprecipitation

Myc-tagged proline-rich protein 19 (PRR19) plasmids and two Flag-tagged CNTD1 versions (including CNTD1 full-length and truncated versions) were co-transfected into the HEK-293T cell line. Cells were then harvested in the IP-lysis buffer (Beyotime, P0013) after 48 h of transfection. For immunoprecipitation input samples, the lysates were spun at 10,000 *g* for 10 min. Anti-Myc Magnetic Beads (Beyotime, P2118) were added to the cell lysate and incubated at 4 °C overnight. The bead-bound proteins were eluted using an SDS loading buffer (Servicebio, G2075) and boiled at 100 °C for 10 min for Western blotting analysis. anti-Flag monoclonal antibody (CST, 14793) and anti-Myc monoclonal antibody (Proteintech, 10828-1-AP) were diluted at 1:2000. Horseradish peroxidase-conjugated goat anti-rabbit (CST, 7074) and horseradish peroxidase-conjugated goat anti-mouse (CST, 7076) secondary antibodies were diluted at 1:5000.

## Results

### Clinical characteristics of the infertile DOR patient

The infertile patient was 23 years old and had no history of pelvic surgery, infection, radiotherapy, or chemotherapy. Hormone measurements of the patient showed declined anti-müllerian hormone levels of 0.63 ng/mL and follicle-stimulating hormone levels of 8.18 mIU/mL. The patient underwent three cycles of controlled ovarian hyperstimulation for *in vitro* fertilization (IVF). Transvaginal ultrasound scan revealed AFC of less than 5 in bilateral ovaries during the three IVF cycles. However, no more than two available oocytes were retrieved each cycle, and no available embryo was obtained during three IVF cycles. Karyotyping and fragile X mental retardation 1 (*FMR1*) pre-mutation analysis of the proband were normal. The clinical characteristics of the patient were summarized in Table 1.

**Table 1:** Phenotypes and investigation of gonadal function in patient with homozygous mutation in *CNTD1*.

Characteristics			
Fem*ale age(years)*	*2*3		
*Karyotype*	*46, XX*		
*FMR1 repeats*	*Normal*		

***Basal hormones***		***Reference***	

*AMH (ng/ml)*	*0.63*	*2–6.8*	
*FSH (mIU/mL)*	*8.32*	*3.2–10.0*	
*LH (mIU/mL)*	*1.77*	*1.80–11.78*	
*E*_*2*_*(pmol/L)*	*39*	*21–251*	
*Prog (nmol/L)*	*0*.*3*	*<0.1–0.3*	
*PRL (ng/mL)*	*21.89*	*5.18*–*26.53*	

***Follicular responses for IVF cycles***	***1***	***2***	***3***

*Antralfolliclecountt(AFC)*	*3*	*4*	*3*
*No. of follicles (≥*14 mm*) on day of HCG administration*	*2*	*2*	*1*

FSH, follicle-stimulating hormone; LH, luteinizing hormone; E2, Estradiol; Prog, Progesterone; PRL, Prolactin.

### A homozygous splicing mutation of *CNTD1* was identified in the patient

**Figure 1:**
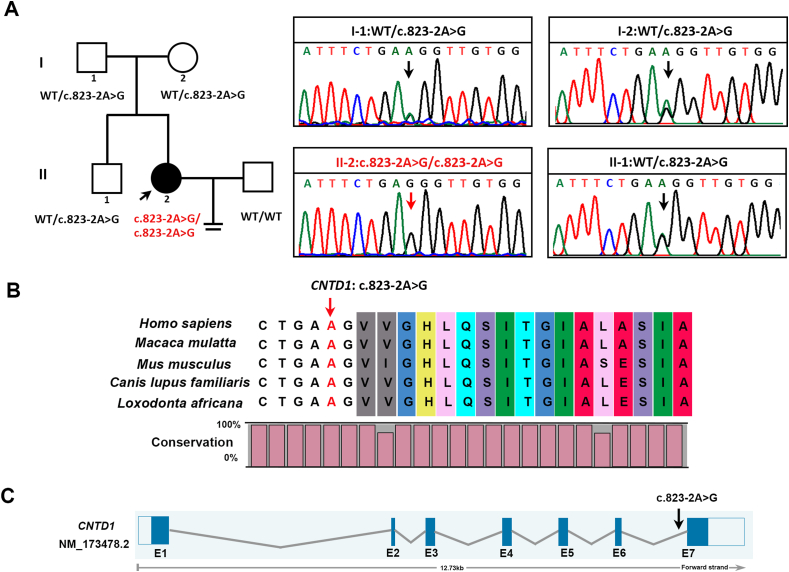
Identification of the pathogenic splicing mutation of *CNTD1* from the pedigree of diminished ovarian reserve. **(A)** Pedigree of the affected family. Family members are designated by Arabic numerals. The squares denote male family members; the circles denote female family members; the arrow denotes the proband; the solid symbols represent affected family members; the open symbols represent unaffected family members; and the equal signs denote infertility. Sanger sequencing was used to validate the genotypes of the proband and the family members, and representative chromatograms are shown. Individuals who are heterozygous for the *CNTD1* mutation (NM_173478.3: c.823-2A > G) show overlapping A and G peaks (I-1, I-2, II-1, parents, and brother of the proband). An individual homozygous for the *CNTD1* mutation (NM_173478.3: c.823-2A > G) has a single G peak (II-2, the proband). **(B)** This *CNTD1* mutation (NM_173478.3: c.823-2A > G) is located at a highly conserved region among multiple species, including *Macaca mulatta*, *Mus musculus*, *Canis lupus familiaris*, and *Loxodonta Africana*. **(C)** Schematic diagram of the location of the genetic variation in *CNTD1* at the genomic level. *CNTD1* is encoded on chromosome 17:42798785–42811587 (NCBI38/hg19); the full-length of human *CNTD1* mRNA comprises seven exons; the identified *CNTD1* homozygous mutation is located in intron 6 adjacent to exon 7 (NM_173478.3: c.823-2A > G).

Variants identified by whole-exome sequencing meeting the following criteria were considered for further validation: i) Mutation type: have effect on protein sequence (nonsense, missense, splicing-site variants, or coding indels); ii) Population databases: have minor allele frequencies (MAF) under 0.0001 among large population databases; iii) Pathogenicity assessment: predicted to be deleterious by pathogenicity predictive tools. Then, a homozygous splicing mutation of *CNTD1* (NC_000017.11 (NM_173478.3): c.823-2A > G) was detected in the patient by whole-exome sequencing. Sanger sequencing of DNA samples from the family members showed that the unaffected brother (II-11) and parents of the proband (I-1 and I-2) carried the *CNTD1* heterozygous mutation, which revealed the autosomal recessive mode of inheritance (Fig. 1A). This *CNTD1* mutation is located at a highly conserved region among multiple species (Fig. 1B). The full-length human *CNTD1* mRNA (NM_173478.3) comprises seven exons, and the identified *CNTD1* homozygous mutation is located in the intron 6 adjacent to exon 7 (Fig. 1C).

### *CNTD1* mutation leads to aberrant splicing

**Figure 2:**
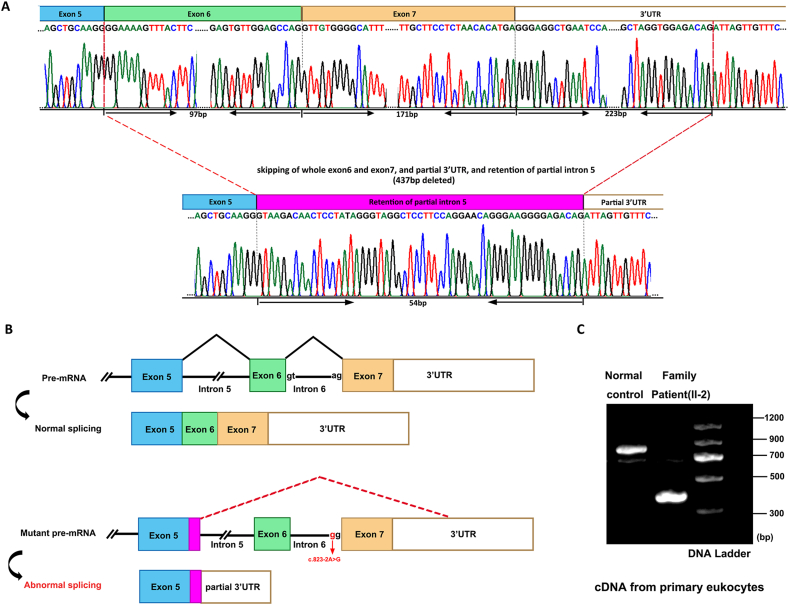
The homozygous *CNTD1* mutation leads to aberrant splicing. **(A)** Through an *in vivo* study in the proband's primary leukocytes, Sanger sequencing of the normal and mutated transcripts resulting from the homozygous *CNTD1* splicing mutation was performed. The *CNTD1* splicing mutation results in retention of partial intron 5 with 54 bp, and skipping of exon 6 with 97 bp, exon 7 with 171 bp, and partial 3′UTR with 223 bp, which disrupts the reading frame. **(B)** The splicing patterns are depicted for patient (II-2) carrying the *CNTD1* c.823-2A > G mutation and the healthy control samples between exon 5 and UTR. **(C)** Agarose gel electrophoresis showed the reverse transcription PCR analysis of mRNA extracted from the peripheral blood of the patient (II-2) carrying the *CNTD1* c.823-2A > G mutation and a healthy control. It also confirmed the skipping fragments caused by the *CNTD1* splicing mutation.

This identified *CNTD1* splicing mutation was not reported in large population databases, including gnomAD. In addition, it was also absent in our in-home database of 200 control subjects with normal ovarian reserve. Multiple prediction tools for splicing mutation, including RDDC (Rare Disease Data Center), HSF (Human Splicing Finder), and SpliceAI, indicated that this *CNTD1* splicing mutation might alter the normal splicing with high probability (Table 2). To validate the splicing result, we performed the direct sequencing of the transcripts of the sample from the proband, which showed that the *CNTD1* splicing mutation resulted in retention of partial intron 5 with 54 bp, and skipping of exon 6 with 97 bp, exon 7 with 171 bp, and partial 3′UTR with 223 bp, which disrupted the reading frame (Fig. 2A and B). Agarose gel electrophoresis also confirmed the skipping fragments of approximately 437 bp in length caused by the *CNTD1* splicing mutation (Fig. 2C). Minigene assay *in vitro* also demonstrated that this mutation caused an aberrant transcript by inducing the skipping of the exon and 5′UTR (Fig. S1).

**Table 2:** The prediction results of the *CNTD1* splicing mutation c.823-2A > G

*In silico* Algorithms	Acceptor Site Prediction Results
RDDC^SC^	Predicted to cause alterative splicing acceptor (Prediction score = 0.9988)
SpliceAI	Predicted to cause acceptor loss (Score = 0.91)
HSF	Predicted to cause alterative splicing acceptor

Note.

RDDC^SC^ (The Rare Disease Data Center, RNA Splicer) (https://rddc.tsinghua-gd.org/).

SpliceAI: https://spliceailookup.broadinstitute.org/.

HSF(Human Splicing Finder): https://www.genomnis.com/access-hsf.

### *Cntd1* knockout mice displayed prematurely depleted follicular pools

**Figure 3:**
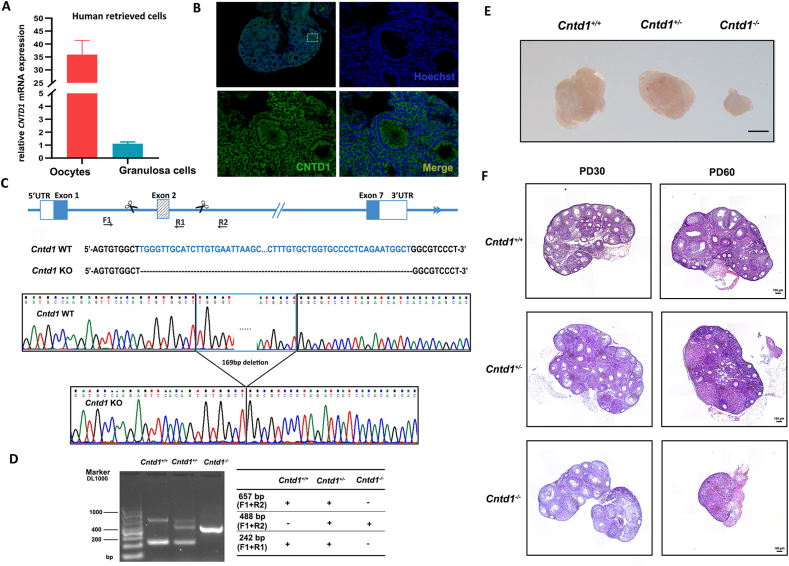
*CNTD1* showed expression in oocytes, and *Cntd1* knockout mice displayed prematurely depleted follicular pools. **(A)** The quantitative PCR in human germinal vesicle (GV) oocytes and human granulosa cells (hGCs) obtained from patients who underwent IVF treatment showed that CNTD1 was highly expressed in oocytes. **(B)** Immunofluorescence analysis showed that the CNTD1 was expressed in follicles. Immunofluorescence staining for CNTD1 was shown in green, and the cell nuclei were counterstained with DAPI and shown in blue from the mouse ovary sections. **(C)** Strategy for the generation of *Cntd1* knockout mice; it caused the deletion of *Cntd1* exon 2 and a frameshift mutation by CRISPR/Cas9 technology. Wild-type *Cntd1* mice were denoted as *Cntd1*^*+/+*^. Heterozygous *Cntd1* knockout mice were denoted as *Cntd1*^*+/−*^. Homozygous *Cntd1* knockout mice were denoted as *Cntd1*^*−/−*^. Sanger sequencing confirmed the creation of the deletion from the mouse genomic DNA. The primers used for genotyping the wild-type and *Cntd1* knockout mice are denoted. **(D)** Agarose gel electrophoresis of the PCR products obtained from genomic DNA of wild-type and *Cntd1* knockout mice. **(E)** The size of ovaries from *Cntd1*^*−/−*^ females was dramatically reduced compared with the control ovaries at postnatal day 60 (PD60). **(F)** The follicles at different developmental stages were observed in *Cntd1*^*+/+*^ ovaries. *Cntd1*^*−/−*^ mouse ovaries showed that there were normal follicles at different developmental stages during PD1 to PD30, but no developing follicle was observed at PD60 when compared with the ovaries from *Cntd1*^*+/+*^ and *Cntd1*^*+/−*^ mice, which indicated prematurely depleted follicular pools in *Cntd1*^*−/−*^ mice.

We investigated *CNTD1* expression patterns in ovaries and follicles based on the single-cell RNA-sequencing data from human ovaries previously, which showed notably expression of CNTD1 in human oocytes and granulosa cells.^17^ Further, through the quantitative PCR expression analysis from human germinal vesicle oocytes and human granulosa cells obtained from patients receiving IVF treatment, we verified that *CNTD1* was highly expressed in adult human oocytes (Fig. 3A). Then, the expression pattern of *CNTD1* in mouse ovaries and oocytes was analyzed by immunofluorescence, and we observed that *CNTD1* was predominantly expressed in follicles at adult age (Fig. 3B). Subsequently, the *Cntd1* knockout mice was generated, involving the deletion of exon 2 and leading to a frameshift mutation by CRISPR/Cas9 technology (Fig. 3C). Amplifying the targeted knockout region of *Cntd1* using PCR primers spanning exon 2 confirmed the knockout efficiency and genotyping (Fig. 3D). The homozygous *Cntd1* knockout mice (*Cntd1*^*−/−*^) were viable and showed no obvious developmental defects in adults. No obvious ovarian morphological abnormal was observed at the timepoints of postnatal day 1 to postnatal day 30. However, by the adult stage to postnatal day 60, the size of ovaries from homozygous knockout females was dramatically reduced compared with the *Cntd1* wild-type (*Cntd1*^*+/+*^) and heterozygous ovaries (*Cntd1*^*+/−*^) (Fig. 3E). The histological analysis of *Cntd1*^*−/−*^ mice ovaries showed that there were normal follicles at different developmental stages during postnatal day 1 to postnatal day 30, but no developing follicle was observed at postnatal day 60 comparing with the ovaries from *Cntd1*^*+/+*^ and *Cntd1*^*+/−*^ mice, which indicated premature depleted follicular pools in *Cntd1*^*−/−*^ mice (Fig. 3F).

### Truncating *CNTD1* mutation abolished interaction with its meiosis partner PRR19

**Figure 4:**
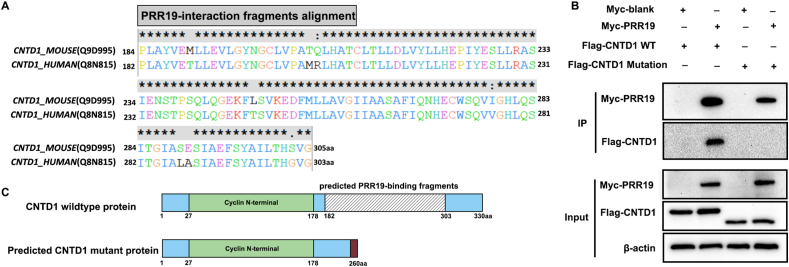
Truncating *CNTD1* mutation abolished interaction with its meiosis partner PRR19. **(A)** The C-terminal amino acid sequence of CNTD1 between amino acids 184–305 is essential to mediate CNTD1–PRR19 interaction in mice. Sequence alignment between the human and mice showed a highly homologous region within the PRR19-interaction amino acid fragment between the human and mice. **(B)** Immunoprecipitation confirmed the interaction between the full-length wild-type CNTD1 and PRR19. By contrast, PRR19 and truncated CNTD1 were barely immunoprecipitated, demonstrating that the mutation affected their interaction, potentially affecting crossover formation during meiosis. **(C)** Schematic representation of the *CNTD1* truncating mutation disrupts the CNTD1–PRR19 interaction.

Meiosis requires CNTD1's interaction with the meiosis-specific protein PRR19. The C-terminal amino acid sequence of CNTD1 is essential to mediate such interaction in mice.^19^ By performing the sequence alignment between the human and mice, we observed a highly homologous region within PRR19-interation amino acid fragment between the human and mice (Fig. 4A). Based on the abnormal splicing result from *CNTD1* mutation, to test whether the truncated *CNTD1* with translation loss of exon 7 will affect the interaction with *PRR19*, the co-expression of MYC-tagged *PRR19* and the two FLAG-tagged *CNTD1* versions (including *CNTD1* full length and truncated version) in HEK-293T cell lines were performed. HEK-293T cell has almost no expression of endogenous CNTD1 nor PRR19 (www.proteinatlas.org/). Immunoprecipitation confirmed the interaction between the full-length wild-type CNTD1 and PRR19. However, the truncated CNTD1 and PRR19 were barely immunoprecipitated. It showed that this *CNTD1* mutation disrupted the CNTD1's interaction with PRR19 significantly, which potentially affected the crossover formation during meiosis (Fig. 4B). Thus, given the impact of the mutation on *CNTD1* pre-mRNA splicing and the immunoprecipitation result, we proposed that the splicing mutation resulted in the truncating CNTD1 and disrupted its interaction with its meiotic partner PRR19 (Fig. 4C). Overall, graphical representation of the identification of *CNTD1* mutation as a novel genetic cause for DOR is showed in Figure S2.

## Discussion

DOR is a prevalent ovarian disorder in women of reproductive age; it is associated with a poor fertility outcome and poses a major challenge in assisted reproductive technology.^20^ The understanding of the etiology of DOR is still largely unknown, and the pathogenic mutations responsible for this condition remain to be discovered. Discovering underlying genetic causes for DOR is essential to improve the diagnosis and help individualize treatments in patients.^21^ Recently, *CNTD1* was reported to play a crucial role during meiosis in mice.^7^^,^^11^ However, *CNTD1* has not been reported in human reproductive diseases. Here, we firstly describe a homozygous *CNTD1* splicing mutation (c.823-2A > G) responsible for the phenotype in the DOR patient. A CRISPR/Cas9-mediated *Cntd1* knockout mouse line was also generated and showed a consistent phenotype with the DOR patient.

*CNTD1* was a crucial gene for meiotic crossover formation. Meiotic crossover mutations play an important role in ovarian development. In mice, defects in meiotic crossover-related genes have been revealed to cause the ovarian phenotype, such as sporulation 11 (*Spo11*),^22^ mutS homolog 4 (*Msh4*),^23^ ring finger protein 212 (*Rnf212*),^24^ and Human enhancer of invasion 10 (*Hei10*).^25^ However, the human genetic evidence of these meiotic crossover-related genes participating in human ovarian pathogenesis was still insufficient. In our study, we reported the phenotype of DOR in a young patient with a homozygous *CNTD1* mutation, and we also revealed the phenotype of *Cntd1* knockout female mice as dramatically reduced ovarian size and prematurely depleted follicular pools. It is consistent with the phenotype caused by other meiotic crossover-related genes, for example, the *Msh*5 knockout female mice were also characterized by progressive atrophic ovaries, oocyte loss, and subinfertility.^26^ As we know that the available ovarian follicle pool determines the female ovarian reserve, and it is established before birth when the oocyte is arrested at the diplotene stage and surrounded by a layer of granulosa cells to form the primordial follicles. Insufficient or excessive activation of primordial follicles would cause accelerated depletion of the follicle pool, leading to DOR and premature ovarian insufficiency.^27^ Hence, this study also highlighted the importance of meiotic crossover genes in preserving the human female ovarian reserve.

The identified *CNTD1* homozygous mutation is located within the acceptor splice site in the intron, and we confirmed the result of the splicing mutation in the patient's leukocytes, which leads to retention of partial intron 5, and skipping of exon 6, exon 7, and partial 3′UTR, thus causing the production of a C-terminally truncated protein. Previous study identified the C-terminal part of mouse CNTD1 protein between positions 184–305 covering the cyclin-box domain as the PRR19-binding domain, which is required for the interdependent partnership between the two proteins and for enabling the meiosis crossover formation.^19^ The CNTD1 protein is highly conserved between humans and mice, with the human harboring the conserved cyclin-box domain between regions of 182–303 amino acids. In this study, we also validated that the truncation induced by the *CNTD1* splicing mutation strongly abolished this interaction. Thus, it indicates the conserved and essential role of CNTD1 partnership in meiosis, and it also suggests the deleterious effect of *CNTD1* mutations with the location of cyclin-box domain. Thus, it was revealed that the identified *CNTD1* mutation caused the production of the truncated CNTD1 protein by inducing aberrant splicing. *CNTD1* mutation may disrupt the meiosis crossover formation by failing to interact with its essential meiotic crossover partner of PRR19. Crossover is critical for meiotic chromosome segregation in oocytes.^28^ *CNTD1* mutation may result in chromosome mis-segregation to cause oocyte aneuploidy, and then checkpoint kinases monitoring oocyte quality were activated, triggering extensive follicular apoptosis and premature depletion of follicular pools.

Furthermore, further investigation was needed to elucidate the underlying pathogenesis mechanism of DOR caused by *CNTD1* deficiency. Moreover, more reports of *CNTD1* mutations are needed, which can help expand the mutation spectrum and enhance our understanding of its role during human fertility.

In conclusion, this study provides the first and direct evidence that *CNTD1* mutation leads to DOR in humans. Further functional experiments verified the pathogenicity of *CNTD1* mutation and suggested the critical role of *CNTD1* in human reproduction. These fundings will facilitate the genetic diagnosis and counselling for the DOR patients, and provide a better understanding of the genetic basis of human ovarian diseases.

## CRediT authorship contribution statement

**Liwei Sun:** Writing – original draft, Investigation, Funding acquisition. **Yi Chen:** Writing – original draft, Investigation. **Keya Tong:** Investigation. **Weiwei Liu:** Investigation. **Bei Liu:** Investigation. **Yifan Wang:** Investigation. **Guoning Huang:** Project administration, Conceptualization. **Jingyu Li:** Writing – review & editing, Supervision, Funding acquisition, Conceptualization.

## Ethics declaration

This study was approved by the ethics committee of the Women and Children's Hospital of Chongqing Medical University (2020-RGI-04, 22 June 2020). The written informed consents were obtained from all participants.

## **Funding**

This work was supported by the grants from the National Natural Science Foundation of China (No. 82302083), the Natural Science Foundation of Chongqing, China (No. CSTB2025NSCQ-GPX0281), the National Reserve Talents Program in the Health Sector of Chongqing (China) (No. HBRC2024015), and CQMU Program for Youth Innovation in Future Medicine (China) (No. W0207).

## Conflict of interests

The authors declared no conflict of interests.
