# Supplementary material for: Identification of the homozygous truncating mutation in CNTD1 as a novel genetic cause of diminished ovarian reserve
Source: Genes Dis. 2025 Oct 24;13(5):101900. doi: 10.1016/j.gendis.2025.101900 (PMC13285272; doi:10.1016/j.gendis.2025.101900)
Supplement: Multimedia component 1 [file mmc1.docx]

**Figure S1** Minigene assay verified the exon skipping induced by the *CNTD1* pathogenic mutation. **(A)** Representation of the human *CNTD1* gene and its mutated form (from Ensembl, reference transcript, NM_173478.3). **(B)** Sanger sequencing of the normal and mutated transcripts resulting from the *CNTD1* mutation. **(C)** Agarose gel electrophoresis showed the reverse transcription PCR analysis of mRNA extracted from transfected cells with the *CNTD1* c.823-2A>G mutation. **(D)** Schematic representation of the normal splicing (upper panel) and the exon 7 skipping (lower panel) induced by the mutation. The aberrant splicing was detected by *in vitro* minigene assay.

**Figure S2** Schematic workflow for identifying the homozygous truncating mutation in *CNTD1* as a novel genetic cause of DOR. First, a homozygous mutations in *CNTD1* was detected in a DOR patient by WES and validated by Sanger sequencing in the pedigree. Subsequently, bioinformatic analyses including (pathogenicity prediction, population database, and cross-species conserved analysis) supported the deleterious effect of the identified mutation. Finally, a CRISPR/Cas9-mediated *Cntd1* knockout mouse model replicated the DOR phenotype, and functionally verifying the crucial role of *CNTD1* in ovarian function.
