# Supplementary figures and images for: Identification of the homozygous truncating mutation in CNTD1 as a novel genetic cause of diminished ovarian reserve
Source: Genes Dis. 2025 Oct 24;13(5):101900. doi: 10.1016/j.gendis.2025.101900 (PMC13285272; doi:10.1016/j.gendis.2025.101900)

A

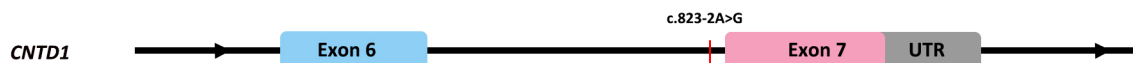

B

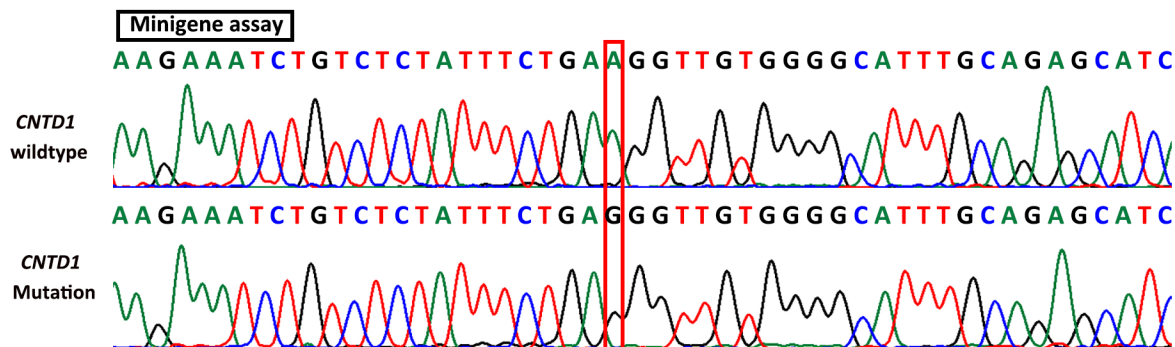

C

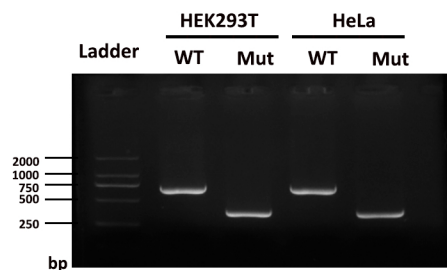

D

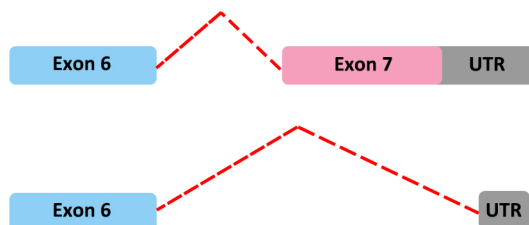

E

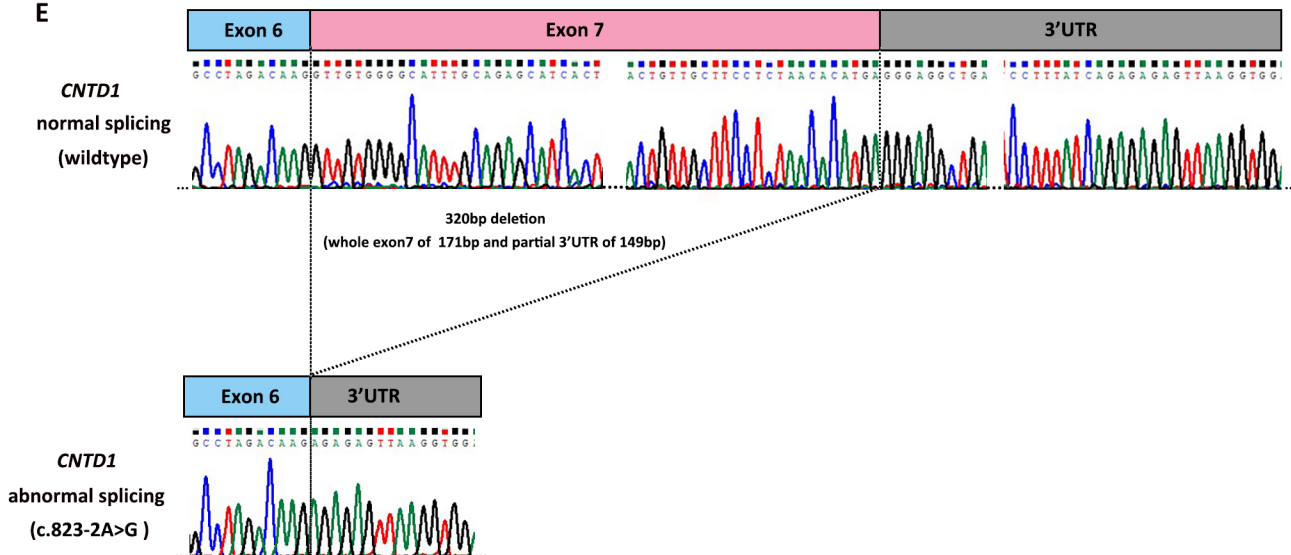

Supplement: Multimedia component 2 [file mmc2.pdf]
