# Supplementary material for: Identification of the homozygous truncating mutation in CNTD1 as a novel genetic cause of diminished ovarian reserve
Source: Genes Dis. 2025 Oct 24;13(5):101900. doi: 10.1016/j.gendis.2025.101900 (PMC13285272; doi:10.1016/j.gendis.2025.101900)
Supplement: Multimedia component 3 [file mmc3.pdf]

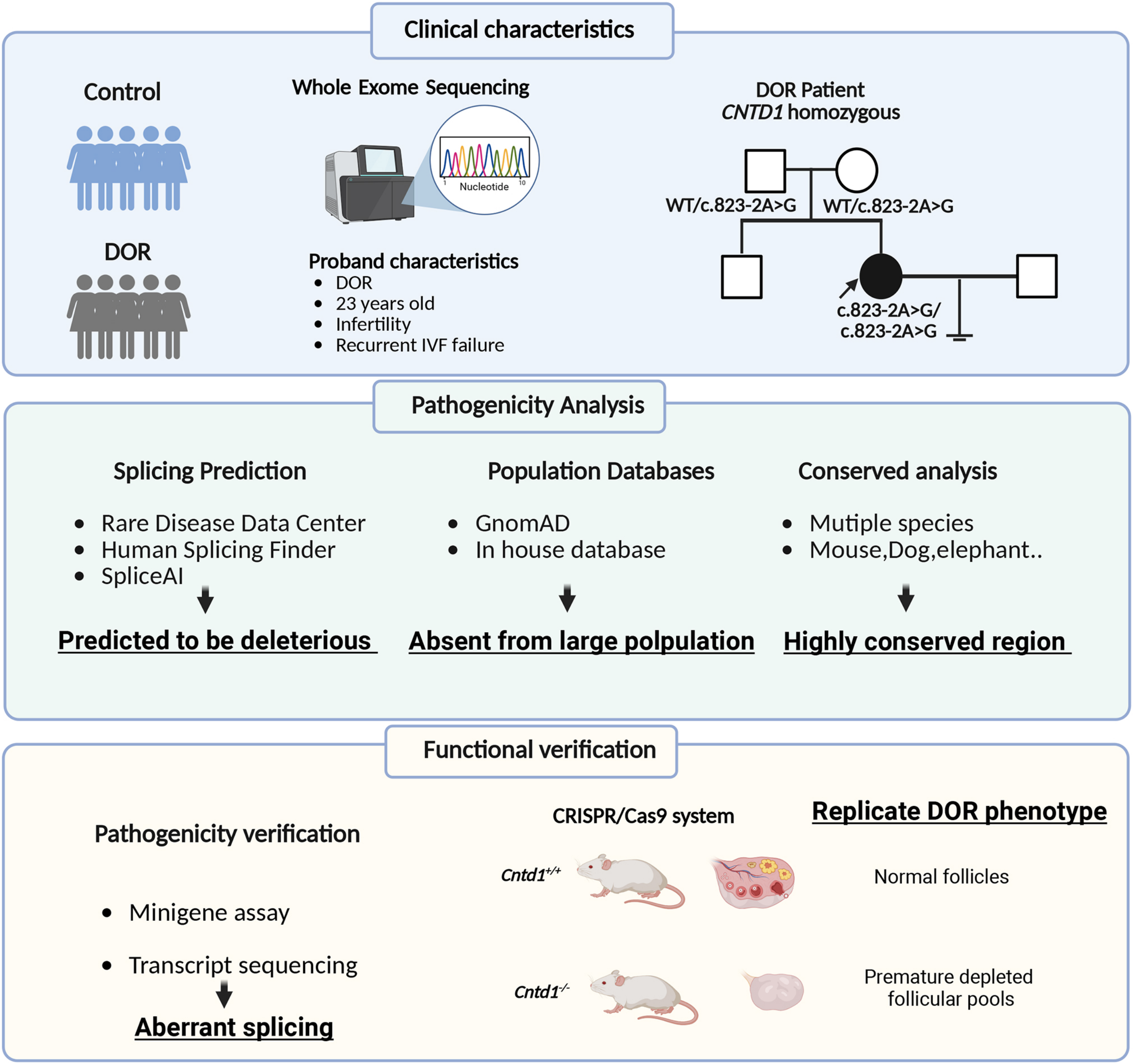

Clinical characteristics
Control
DOR
Whole Exome Sequencing
Nucleotide
Proband characteristics
DOR
23 years old
Infertility
Recurrent IVF failure
DOR Patient
CNTD1 homozygous
WT/c.823-2A>G
WT/c.823-2A>G
c.823-2A>G/
c.823-2A>G
Pathogenicity Analysis
Splicing Prediction
Rare Disease Data Center
Human Splicing Finder
SpliceAI
Predicted to be deleterious
Population Databases
GnomAD
In house database
Absent from large polpulation
Conserved analysis
Mutiple species
Mouse,Dog,elephant..
Highly conserved region
Functional verification
Pathogenicity verification
Minigene assay
Transcript sequencing
Aberrant splicing
CRISPR/Cas9 system
Replicate DOR phenotype
Cntd1+/+
Normal follicles
Cntd1-/-
Premature depleted follicular pools
